# Dual functions of the ΔNp63-miR-141-3p-YAP1 regulatory axis in cervical cancer progression are dependent on histological subtype

**DOI:** 10.1038/s41598-025-07237-6

**Published:** 2025-07-02

**Authors:** Somayeh Panahi-Moghadam, Majid Sadeghizadeh, Shirin Farivar, Faezeh Vakhshiteh

**Affiliations:** 1 Department of Molecular Genetics, Faculty of Biological Sciences, Tarbiat Modares University, Tehran, 14115154 Iran; 2 Department of Cell and Molecular Biology, Faculty of Life Sciences and Biotechnology, Shahid Beheshti University, Tehran, 1983963113 Iran; 3 Oncopathology Research Center, Iran University of Medical Sciences (IUMS), Tehran, 1449614535 Iran

**Keywords:** Cervical cancer, ΔNp63, EMT, miR-141-3p, YAP1, Cancer, Cancer genetics, miRNAs, Molecular biology, Cell biology, Cell adhesion, Cell migration, Cell signalling, Genetics, RNAi

## Abstract

Cervical cancer (CC) poses a significant global health challenge, necessitating the development of novel therapeutic strategies. The interplay between the p63 isoform (ΔNp63), microRNA-141-3p (miR-141-3p), and Yes-associated protein 1 (YAP1) has emerged as a potential area of interest in cancer progression. This study aimed to investigate the functional relationship between the between the p63 isoform (ΔNp63), miR-141-3p and YAP1 in modulating migration, invasion, and epithelial–mesenchymal transition (EMT) in two CC cell lines, CaSki, and HeLa, which are human cervical squamous cell carcinoma (SCC) and adenocarcinoma (ADC) cells, respectively. The Gene Expression Omnibus (GEO) datasets, were utilized to assess the expression profiles of TP63 and YAP1 in the cervical SCC and ADC samples by using the GEO2R tool. The prediction software (miRDIP, miRmap, and TargetScan), and RT-qPCR were used to determine the relationship between genes. Different assays were performed for proliferative, migratory and invasive abilities of cells. The results were confirmed by western blot analysis. The ΔNp63-miR-141-3p-YAP1 axis exhibited distinct, cell-line-specific functions. In HeLa cells, this axis promoted a prometastatic phenotype by upregulating YAP1, leading to increased proliferation, migration, invasion, and EMT. Conversely, in CaSki cells, the same axis demonstrated an antimetastatic function by downregulating YAP1. YAP1 expression was significantly higher in HeLa cells compared to CaSki cells. In HeLa cells, YAP1 expression appeared to be regulated not solely by the upstream ΔNp63-miR-141-3p axis, suggesting its role as a key oncogene in HeLa cell progression. MiR-141-3p demonstrated context-dependent effects, exhibiting both pro- and anti-metastatic activities depending on the specific cell line. These findings highlight the complex and subtype-specific functions of the ΔNp63-miR-141-3p-YAP1 regulatory network in cervical cancer progression. In summary, our data highlights the different functions of ΔNp63-miR-141-3p-YAP1 axis in regulating proliferation, migration and invasion as well as EMT of different cervical cancer cells.

**Supplementary Information:**

The online version contains supplementary material available at 10.1038/s41598-025-07237-6.

## Introduction

Cervical cancer is one of the most common cancers affecting women worldwide, and it is the second most common cause of cancer death^1,2^. Prophylactic vaccines against high-risk HPVs are available, and patients with early-stage CC can be treated with surgery, chemotherapy, and radioimmunotherapy. As large groups of diagnosed CC patients are at intermediate and advanced stages with poor prognoses, the establishment of molecular targeted therapy against invasion and metastasis of CC is urgently needed. Adenocarcinoma (ADC) and squamous cell carcinoma (SCC) are the two main subtypes of CC, accounting for 25% and up to 70%, respectively, of all cases. Growing evidence illustrates that due to a lack of clinical symptoms, increased tendency for metastasis, recurrence, and resistance to chemotherapy, patients with ADC exhibit a worse prognosis and a lower survival rate than patients with SCC^3,4^.

During epithelial-mesenchymal transition (EMT), epithelial cancer cells usually lose their phenotype and exhibit mesenchymal characteristics, which renders tumors less responsive to clinical treatments and more invasive. Dysregulation of EMT-associated genes, such as Twist1, E-cadherin, and N-cadherin, promotes EMT and results in the invasion and metastasis of cervical cancer^5,6^. In 2017, a thorough genomic analysis was carried out on 228 samples of primary cervical cancer tumors. The findings of this analysis highlighted a noteworthy phenomenon: the YAP1 gene underwent significant amplification and experienced copy number (CN) gain in cervical cancer samples exhibiting EMT characteristics, marked by high expression of EMT-related proteins and reduced miR-200a-3p levels, indicating enhanced metastatic potential. Furthermore, the YAP1 protein displayed a distinctive expression pattern in these EMT-associated samples, particularly within the adenocarcinoma-rich subgroup, distinguishing them from other samples. This observation suggests a potentially crucial role for YAP1 in the molecular characteristics of EMT in cervical cancer, emphasizing its significance in cancer progression^7^.

YAP1 (Yes-associated protein 1), a transcriptional co-activator regulated by the Hippo signaling pathway, plays a pivotal role in regulating cell proliferation, survival, and EMT. In the absence of Hippo pathway activation, YAP1 translocates into the nucleus and cooperates with transcription factors such as TEADs to induce the expression of genes associated with cell migration and stemness, thereby promoting mesenchymal traits^8–10^. Overactivation of YAP1 leads to uncontrolled cell proliferation, resistance to apoptosis, progression of EMT and metastasis. Several cancers are associated with YAP1 dysregulation, including liver cancer, breast cancer, and cervical cancer^8,11,12^.

*TP63* gene, a member of the p53 gene family, produces multiple isoforms via alternative splicing and promoter usage, primarily the transactivation (TAp63) and N-terminal truncated (ΔNp63) variants. These isoforms exhibit opposing biological functions: while TAp63 is generally pro-apoptotic and tumor-suppressive, ΔNp63 functions predominantly to maintain epithelial integrity and suppress EMT^13,14^. Specifically, ΔNp63 is highly expressed in basal epithelial cells and contributes to epithelial identity by upregulating adhesion molecules and inhibiting transcriptional repressors of epithelial markers such as claudin 1 and integrins^15,16^.

According to the TCGA study which mentioned previously^7^, p63 isoforms were identified as key modulators of signaling divergence in SCC, influencing the activation or inhibition of downstream targets and potentially affecting tumor behavior and treatment responses. Furthermore, in prostate epithelial cells, increased expression of ∆Np63 has been shown to cause a major shift in gene expression patterns favoring epithelial features^17^. Emerging evidence shows that ΔNp63 can indirectly inhibit EMT through the activation of miRNAs such as the miR-200 family, which are known to suppress ZEB1/2^18^.

The role of the miR200 family, including miR141, in suppressing EMT has been well established^19^. Members of the miR-200 family typically function to maintain the epithelial phenotype by targeting and inhibiting the expression of transcription factors such as ZEB1 and ZEB2^19–21^. An integrated genomics approach in ovarian cancer revealed that p63 directly regulates the activity of the shared promoter of miR-200c/141 and acts as an activator of the expression of these miRNAs^22^. Notably, the specific role of miR-141 can vary among different cancer types^23–25^. Using target prediction tools, such as miRDIP, miRmap and TargetScan, we found that miR-141-3p strongly targets the 3’UTR of YAP1 mRNA. Considering that ΔNp63 and YAP1 are involved in the EMT process in CC and based on their negative correlation in two major histological cervical cancer cell lines, we aimed to investigate the interplay between ΔNp63 and YAP1 in the SCC CaSki and ADC HeLa cell lines. We also investigated whether miR-141 plays an intermediate role between p63 and YAP1.

## Materials and methods

### Bioinformatics analysis

We initially conducted an in silico investigation to compare the expression levels of the *TP63* and *YAP1* genes in SCC and ADC samples. The GSE27388 dataset, sourced from the Gene Expression Omnibus (GEO), was utilized to assess the expression profiles of *TP63* and *YAP1* in the abovementioned samples. The dataset comprised 19 SCC samples with a positive P63 staining status and 9 ADC samples with a negative p63 staining status. The differential expression of p63 and YAP1 between these two groups was determined using the GEO2R tool. To predict strong and very strong targets of miR-141-3p, the bioinformatics tools MicroRNA Data Integration Portal (miRDIP) (http://ophid.utoronto.ca/miRDIP) miRmap (https://mirmap.ezlab.org/) and TargetScan (https://www.targetscan.org/) were used. Among these identified targets, genes associated with EMT were selectively screened. Furthermore, a protein‒protein interaction (PPI) network involving YAP1, p63, miR-141-3p, and key regulators of EMT was constructed using STRING (version 10.5; http://string-db.org/) and visualized through Cytoscape software (version 3.6.1; http://www.cytoscape.org/). Hub genes, representing central nodes in the network, were identified utilizing the CytoHubba plugin based on maximal clique centrality (MCC) metrics.

### Cell culture

The Iranian Biological Resource Center (IBRC) provided us with the human CC cell lines HeLa and CaSki. All cells were cultured in RPMI 1640 (Gibco, USA) or high-glucose DMEM (Gibco, USA) supplemented with 10% fetal bovine serum (FBS) (Gibco, USA), 100 IU/mL penicillin and µg/mL streptomycin (Bioidia, IRAN) at 37 °C in a humidified atmosphere with 5% CO2 at 37 °C.

### Plasmid construction and cell transfection

Short hairpin sequences targeting the ΔNp63 RNA isoform were designed using Web.stanford.edu and inserted into the BamHI-HindIII site of the pRNA-H1/Neo vector. The sense and antisense sequences of the sh-RNAs were as follows: 5′-sh-RNA sense strand: 5′-CGCGGATCCGCGGCAGCATTGTCATTCTTAATACCTGACCCATA-3′; sh-RNA antisense strand: 5′-CCCAAGCTTGGGAAAAAGCAGCATTGTCATTCTTAATATGGGTCAGGTA-3’. The sequences of the miR-141-3p inhibitor and miR-141-3p mimic were as follows: miR-141-3p inhibitor, 5′-CCAUCUUUACCAGACAGUGUUA-3′; miR-141-3p mimic, forward 5′- UAACACUGUCUGGUAAAGAUGG-3′ and reverse 5′-A UCUUUACCAGACAGUGUUAUU-3′; mimic negative control (NC), forward 5′-UUCUCCGAACGUGUCACGUTT-3′ and reverse 5′-ACGUGACACGUUCGGAG AATT-3′; and inhibitor negative control (inh-NC), 5′-CAGUACUUUUGUGUAGUACAA-3′. All of the RNA molecules were obtained from GenePharma (Suzhou, China) and transfected into CaSki and HeLa using Lipofectamine 3000 (Invitrogen, USA). Transfection efficiency was assessed using GFP fluorescence plasmid and FAM labeled oligonucleotides. Cytotoxicity was evaluated 48 h after transfection, using trypan blue staining.

### RNA isolation, cDNA synthesis, and qRT‒PCR

RiboEx reagent (GeneAll, Korea) was used to extract RNA from cultured cell lines. The RNA quality and quantity were evaluated using a Nanodrop device (Thermo Fisher Scientific, USA). The RNA samples were reverse-transcribed to cDNA using an Easy cDNA Synthesis Kit (Parstous, IRAN). The relative expression of the TAp63, ΔNp63, and YAP1 isoforms was measured using SYBR Green 2x Master Mix (ParsTous, IRAN) on an MIC qRT‒PCR thermocycler (Biomolecular Systems, Australia), with the GAPDH gene serving as a housekeeping gene. The cycling procedure involved initiation at 94 °C for 5 min, followed by 35 cycles of 94 °C for 15 s, 64 °C for 20 s and 72 °C for 20 s. Before cDNA synthesis, miR-141-3p and U48 were polyadenylated using *E. coli* poly(A) polymerase (New England Biolabs Inc., USA) according to the manufacturer’s guide. Gene expression levels were compared between samples using the ΔΔCt method.

### Cell proliferation assays

The proliferation ability of the cells was determined by colony formation and MTT assays. Following transfection, CaSki and HeLa cells (5,000 cells/well) were seeded onto 96-well plates with flat bottoms. The culture medium was refreshed regularly. Cell proliferation was assessed using a colorimetric MTT assay. At defined time points (0, 24, 48, 72, and 96 h), 10 µL of MTT solution (5 mg/mL in PBS, Sigma-Aldrich, USA) was added to each well. The plates were then incubated for 3 h at 37 °C. After incubation, the medium was removed and replaced with 100 µL of DMSO (Sigma-Aldrich, USA) per well to solubilize formazan crystals. The results were assessed using a Universal Microplate Reader (Bio-Tek Instruments, Inc., Winooski, VT, USA) by measuring absorbance at 570 nm. For the colony formation assay, CaSki and HeLa cells were transfected and incubated for 48 h. Then, the cells were trypsinized (Bioidea, Iran) and seeded onto 12-well plates at a density of 400 cells per well. Cells were cultured for 11 days under standard conditions in RPMI 1640 (CaSki cells) or DMEM (HeLa cells) supplemented with 10% FBS. Following fixation with 70% ethanol, colonies were stained with 0.5% crystal violet solution (Sigma‒Aldrich, USA) and visualized and counted.

### Cell invasion and migration assays

A cell invasion assay was performed using 8 μm transwell inserts (SPL, Life Science, South Korea) with 50 µL of Matrigel (Corning, USA) diluted in serum-free medium. Forty-eight hours after transfection, CaSki and HeLa cells were rinsed and incubated in medium lacking serum for 12 h. Then, 50,000 cells from a single-cell suspension were placed in the upper chamber of a transwell plate, and 500 µL of 10% FBS-containing medium was added to the bottom of well as a chemoattractant. After 24 h, the cells that invaded through the membrane and reached the lower chamber, were stained with a crystal violet cell staining solution. For statistical analysis, the stained cells were counted. The same protocol was conducted for cell migration assays, but without Matrigel.

### Western blot analysis

RIPA buffer was used to prepare cell lysates (Roche, Nutley, NJ, USA). For protein quantification, a BCA protein assay kit (Thermo Fisher Scientific, USA) was used. Using SDS-PAGE, protein samples were separated and then transferred to PVDF membranes (Millipore, USA). To minimize nonspecific binding, the membranes were blocked with 2% nonfat dry milk for 1:15 h and incubated with primary antibodies overnight at room temperature. Primary and secondary antibodies (Santa Cruz Biotechnology, USA) used in this study are: mouse monoclonal anti-Vimentin (sc-373717, 1:300), mouse monoclonal anti-Ecadherin (sc-21791, 1:300), mouse monoclonal anti-YAP1 (sc-101199, 1:300), mouse monoclonal anti-glyceraldehyde 3-phosphate dehydrogenase (GAPDH) (sc-32233, 1:300), mouse IgGκ HRP (sc-516102; 1:1000), and mouse anti-rabbit IgG-HRP (sc-2357, 1:1000). Next, detection of band was performed with an enhanced chemiluminescence (ECL) kit and the immunoblot signals were quantified with ImageJ software.

### Statistical analysis

Three independent replicates of experiments were performed to assess the reliability of each assay and data were presented as mean ± standard deviation (SD). Data were analyzed using GraphPad Prism (version 8.4.3). One-way ANOVA followed by Tukey’s multiple comparison test or Student’s t-test were used to determine the level of statistical significance.

## Results

### In silico analysis of the differential expression of YAP1 and p63 in ADC and SCC

**Fig. 1:**
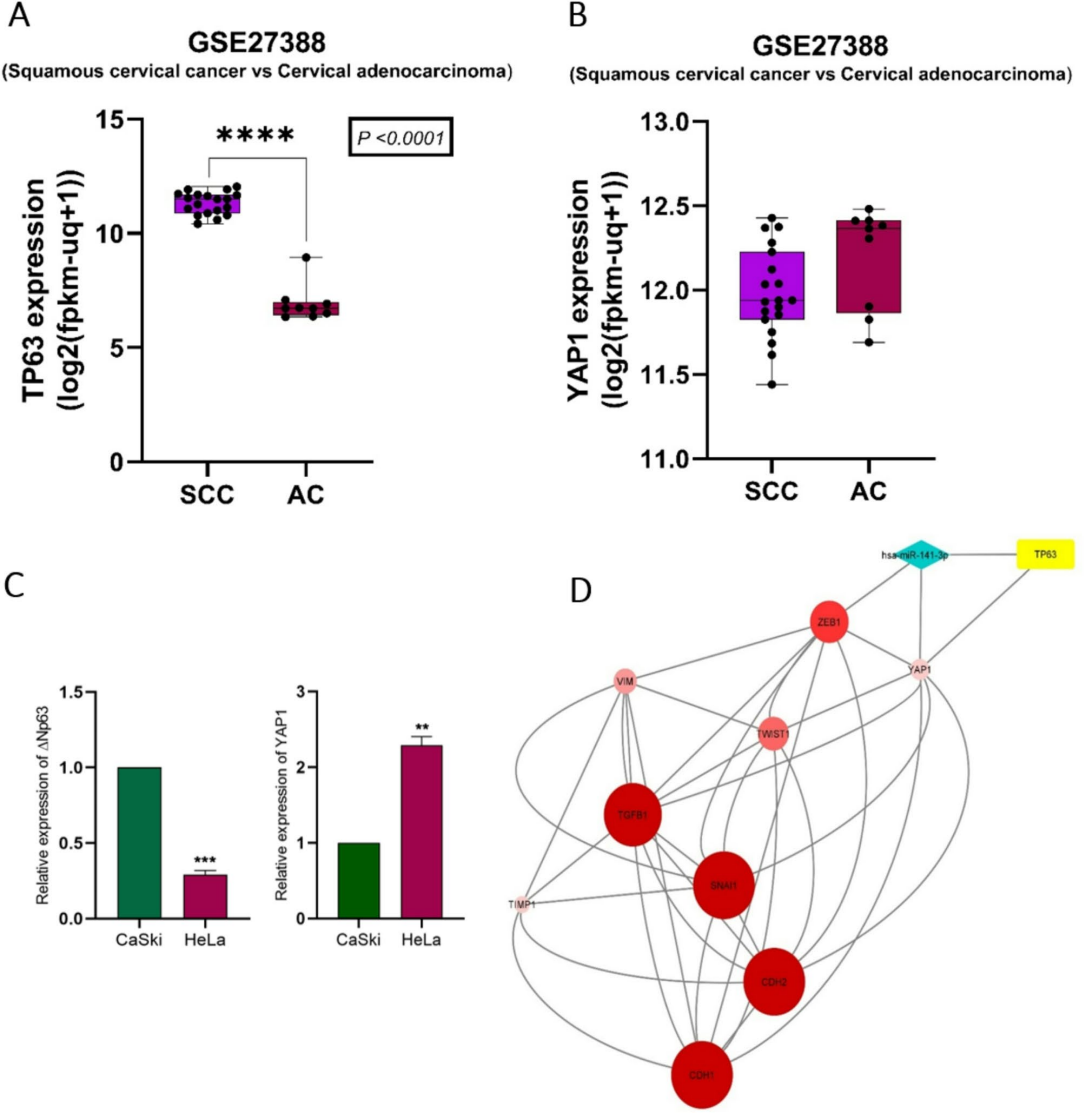
**A**,** B** In silico analysis of TP63 and YAP1 expression patterns in 19 Squamous cell carcinoma (SCC) and 9 Adenocarcinoma (ADC) tissue samples. Expression levels of TP63 and YAP1 in patients with SCC compared to patients with ADC based on the GSE27388 dataset (****P < 0.0001). **C** Expression levels of ΔNp63 and YAP1 in CaSki and HeLa cells. ΔNp63 is a major isoform of *TP63* and our experimental data specifically refer to ΔNp63 expression, while in silico analysis pertains to TP63 as a whole gene. **D** Possible interactions between p63-miR141-3p-YAP1 and key regulators of EMT based on in silico analysis. GAPDH was used as an internal control. ** *P* < 0.01, and *** *P* < 0.001 indicate significant differences.

The microarray dataset (GEO accession number: GSE27388) includes nineteen cervical squamous cell carcinoma (SCC) and nine adenocarcinoma (AC) 10 ~ 16-year-old FFPE samples were profiled using Affymetrix Exon 1.0 ST arrays. The analysis of the data suggested that the expression of *Tp63* in SCC was substantially higher than that in ADC. Furthermore, the expression level of *YAP1* was marginally lower in the SCC than in the ADC (Fig. 1A and B). The results of gene expression studies in Caski and HeLa cell lines confirmed the in silico results, showing higher and lower expression of *Tp63* (*p* < 0.001) and *YAP1* (*p* < 0.01) in Caski cells than HeLa cells (Fig. 1C). miR-141-3p target prediction was then conducted using the mirDIP, miRmap, and TargetScan databases. The results indicate that, within the set of crucial regulators involved in EMT, both ZEB1 and YAP1 are highly significant targets of miR-141-3p. The network depicted in Fig. 1D illustrates the close associations between miR-141-3p, YAP1, p63, and main regulators of EMT. These findings strongly indicate that aberrant expression patterns of miR-141-3p, YAP1, and p63 can impact EMT.

### Dual role of ΔNp63 in regulating proliferation, migration, and invasion in ADC HeLa and SCC CaSki cells

**Fig. 2:**
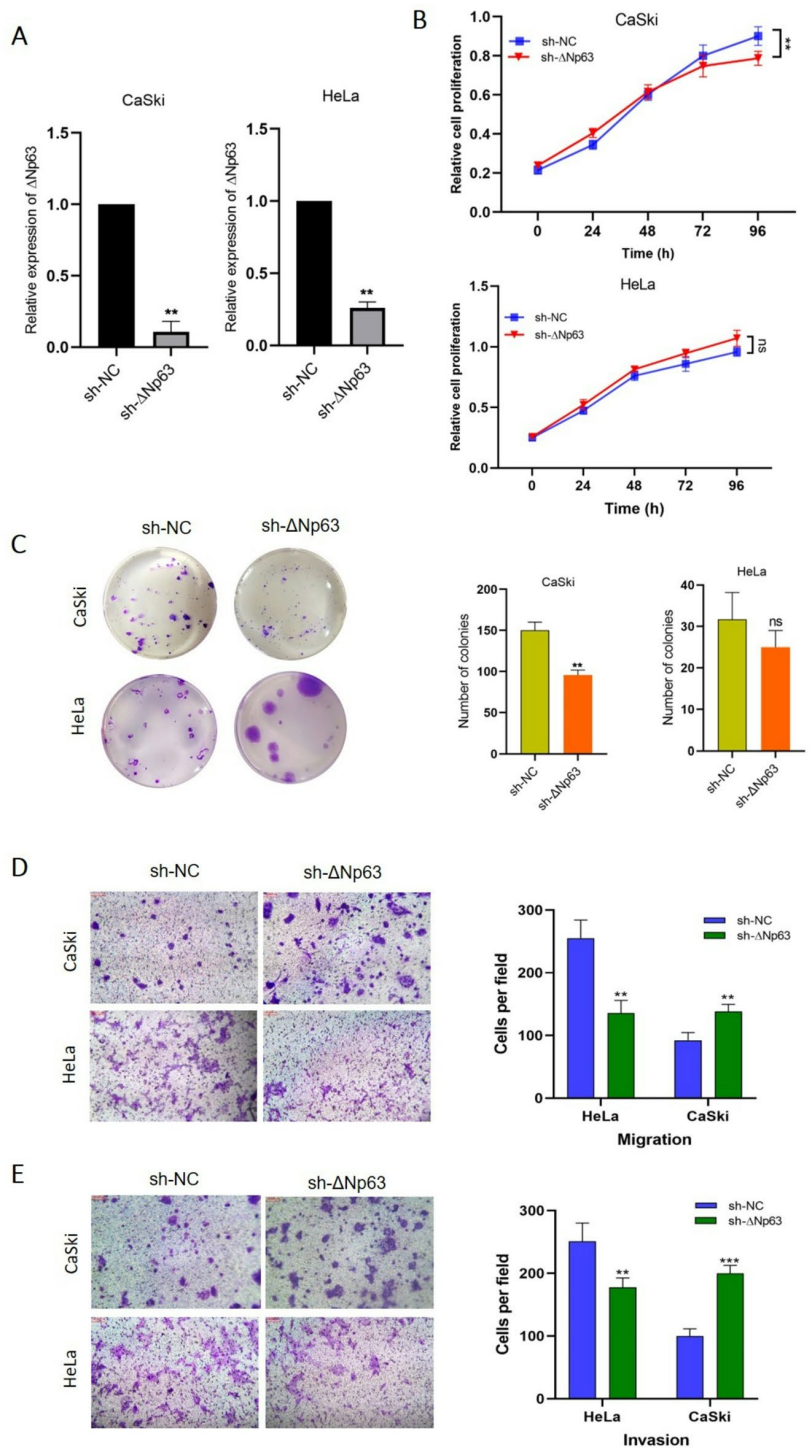
Dual neoplastic features of ΔNp63 knockdown in cervical cancer cell lines **A** ΔNp63 was silenced in CaSki and HeLa cells by transfection with the specific sh-ΔNp63 construct (qRT‒PCR results). An empty vector was used as a control. **B**,** C** After ΔNp63 silencing, proliferation was evaluated through MTT and colony formation assays. **D**,** E** Transwell migration and invasion assays revealed that ΔNp63 regulates the migration and invasion of CaSki and HeLa cells in different manners. ** *P* < 0.01, and *** *P* < 0.001 represent significant differences; ns = not significant vs. control.

The differential expression of the *TP63* and *YAP1* genes in cervical SCC and ADC suggested that p63 and YAP1 play opposing roles in the progression of these two types of cancer (see Fig. 1). The qRT‒PCR results showed that the expression of the TAp63 isoform was undetectable in HeLa and CaSki cells. In addition, several studies have reported that ΔNp63 is the main isoform of p63 that acts as both an oncogene and a tumor suppressor, especially during EMT progression^15,16,26,27^; Therefore, we examined the effect of ΔNp63 downregulation on the proliferation, migration, and invasion capacities of HeLa and CaSki cells. ΔNp63 silencing by sh-RNA was performed in both cell lines. Cells were transfected with either ΔNp63 sh-RNA or empty vector as a negative control. qRT‒PCR revealed an 5 to 8-fold decrease in ΔNp63 expression compared to that in control cells (*p* < 0.01) (Fig. 2A). Next, proliferation assays including MTT and colony formation, were performed to evaluate the impact of ΔNp63 knockdown on cell growth. The results indicated no significant difference between sh-ΔNp63 treated and control HeLa cells; however, an increase in colony size was observed. In contrast, in CaSki cells, ΔNp63 knockdown resulted in a decrease in cell proliferation with no difference in colony size between the control and sh-ΔNp63 groups (*p* < 0.01) (Fig. 2B and C).

Transwell assays demonstrated that the migratory and invasive abilities of CaSki cells were significantly increased in the sh-ΔNp63 transfected group compared to the control group. In contrast to CaSki cells, ΔNp63 downregulation decreased the migration and invasion of HeLa cells (*p <* 0.01 and *p <* 0.001) (Fig. 2D and E). Previous studies have also highlighted the contradictory tumor-suppressing or oncogenic roles of p63 in different cancer types^16,27–29^. Our results confirmed the dual role of ΔNp63 in various cervical cancer cell lines with distinct origins.

### Pro- and anti-metastatic effects of miR-141-3p in cervical cancer cell lines

**Fig. 3:**
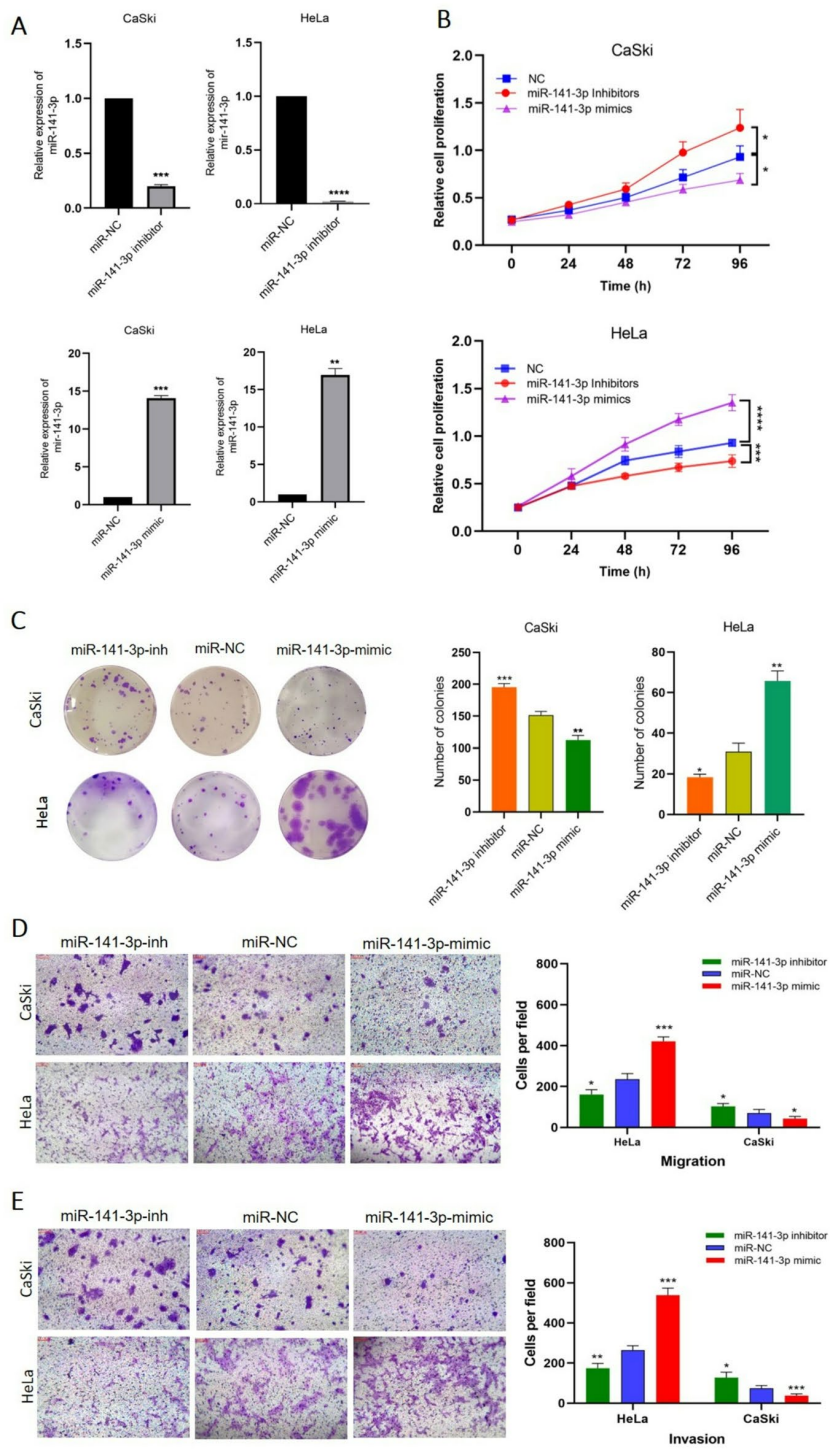
Suppressive and oncogenic roles of miR-141-3p on CaSki and HeLa cells. **A** Efficiency of inhibitor/mimic transfection was evaluated by qRT‒PCR. NC-oligos were used as a negative control. **B**,** C** Following either miR-141-3p silencing or overexpression, both MTT and colony formation were used to assess cell proliferation. **D**,** E** Transwell assays indicated that ΔNp63 regulates the migratory and invasive properties of CaSki and HeLa cells in different manners. * *P* < 0.05, ** *P* < 0.01, *** *P* < 0.001, and **** *P* < 0.0001 indicate significant differences.

In the subsequent step, HeLa and CaSki cells were transfected with miR-141-3p mimics and inhibitors, to evaluate their proliferative, migratory, and invasive abilities. First, the transfection of miR-141-3p inhibitor/mimic was successfully confirmed by qRT‒PCR (Fig. 3A). In HeLa cells, transfection with miR-141 mimic resulted in a significant increase in proliferation rate as showed by MTT and colony formation assays (Fig. 3. B and C). In addition, miR-141-3p mimic transfection significantly increased the migration and invasion of HeLa cells (Fig. 3D and E). However, the impact of these experiments in CaSki cells was opposite to those in HeLa cells, demonstrating that the upregulation of miR-141 led to a decrease in the proliferation (Fig. 3B and C), migration, and invasion of CaSki cells (Fig. 3D and E) compared to the negative controls. Accordingly, downregulation of miR-141-3p by inhibitors suppressed the proliferation (Fig. 3B and C), migration, and invasion of HeLa cells (Fig. 3D and E); however, in CaSki cells, the proliferation, migratory, and invasive rate of inhibitor transfected groups were apparently higher than control groups (Fig. 3B-E). Our data substantiates the opposite roles of miR-141-3p in suppressing and/or promoting invasion in cervical cancer cell lines with distinct origins, as previously demonstrated in other cancer cells^30–32^.

### Effects of ΔNp63 knockdown on cell migration and invasion reversed by miR-141-3p upregulation

**Fig. 4:**
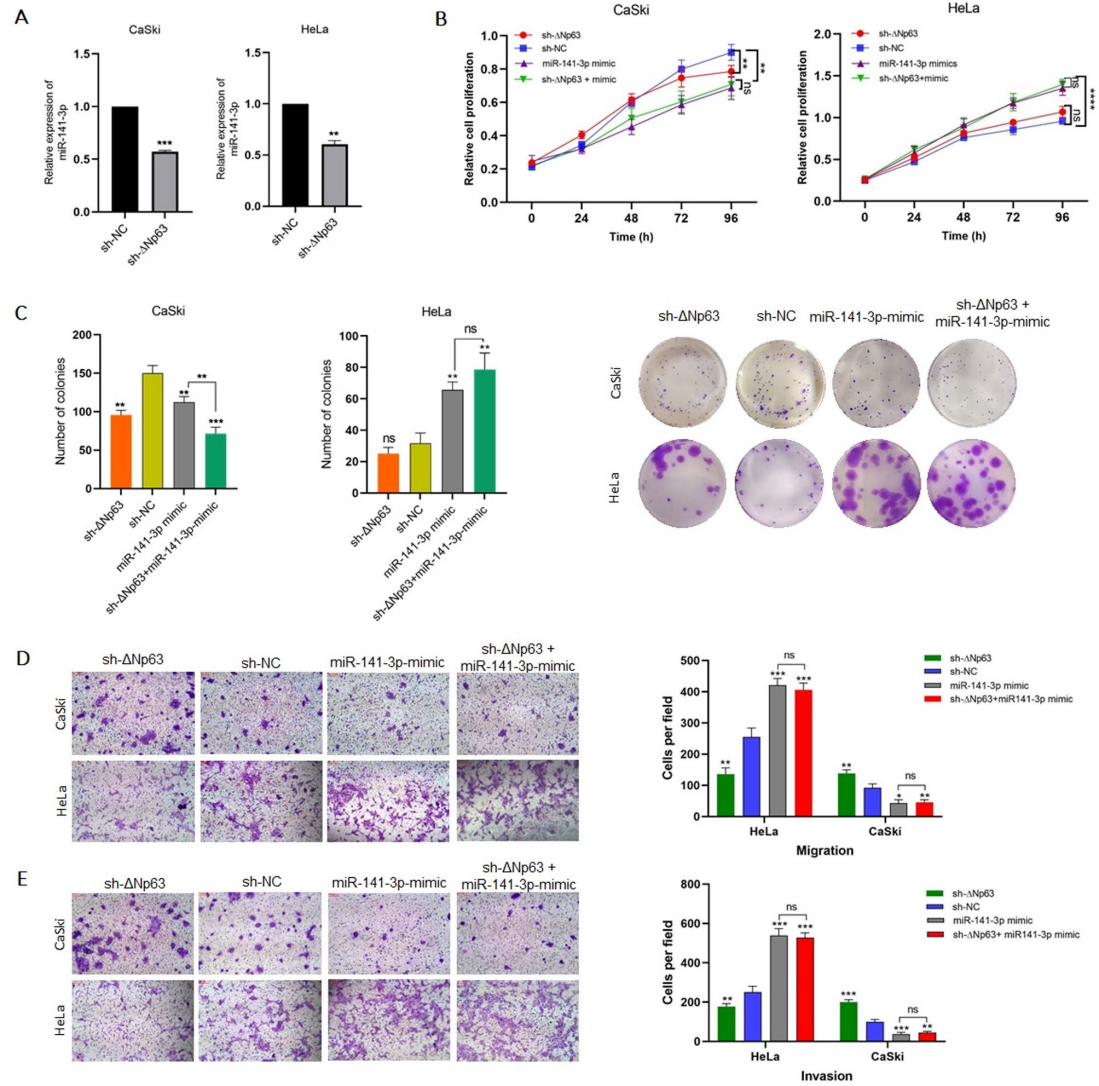
sh-ΔNp63/miR-141-3p mimic co-transfection features are comparable to miR-141-3p mimic single transfection. **A** q-PCR results showed that the expression of miR-141-3p is under the control of ΔNp63. **B**,** C** cell proliferation results of sh-ΔNp63/miR-141-3p mimic co-transfection; MTT performed at time points of 0, 24, 48, 72, and 96 h, and colony formation tracked for 11 days **D**,** E** Transwell assays were used to identify the influence of single and co-transfections on cell migration and invasion. * *P* < 0. 1,* * *P* < 0.01, *** *P* < 0.001, and **** *P* < 0.0001 represent significant differences; ns = not significant vs. control.

In Sect. "Dual role of ΔNp63 in regulating proliferation, migration, and invasion in ADC HeLa and SCC CaSki cells", we described the effects of ∆Np63 downregulation in CaSki and HeLa cells (see Fig. 2). We first investigated the impact of ∆Np63 knockdown on the expression of miR-141-3p (Fig. 4A). We next investigated whether miR-141-3p, as a potential downstream effector of ΔNp63, could influence the cellular phenotypes observed upon ΔNp63 silencing. To this end, rescue experiments were conducted by co-transfecting sh-ΔNp63 and a miR-141-3p mimic into CaSki and HeLa cells. Interestingly, the outcomes of ∆Np63 knockdown was compensated by the increased level of miR-141-3p, which led to decreased proliferative, migratory, and invasive abilities in CaSki cells (Fig. 4B–E). The same experiment in HeLa cells exhibited an increased proliferative and invasive characteristics of these cells (Fig. 4B–E). Data are reported as mean ± SD of three replicates. In most experimental results presented in Fig. 4, the comparison between the miR-141-3p mimic and sh-∆Np63/miR-141-3p mimic groups did not show a statistically significant difference, suggesting that the upregulation of miR-141-3p largely compensates for the effects of p63 knockdown. Overall, these observations confirmed the role of miR-141-3p as a mediator of ∆Np63 function, in such a way that maintaining miR-141-3p at high levels neutralizes the effect of ∆Np63 and indicates that without the mediatory role of miR-141-3p, ∆Np63 cannot exert its tumor suppressor or oncogenic effects in two distinct cell lines.

### ΔNp63 targets YAP1 via miR-141-3p

**Fig. 5:**
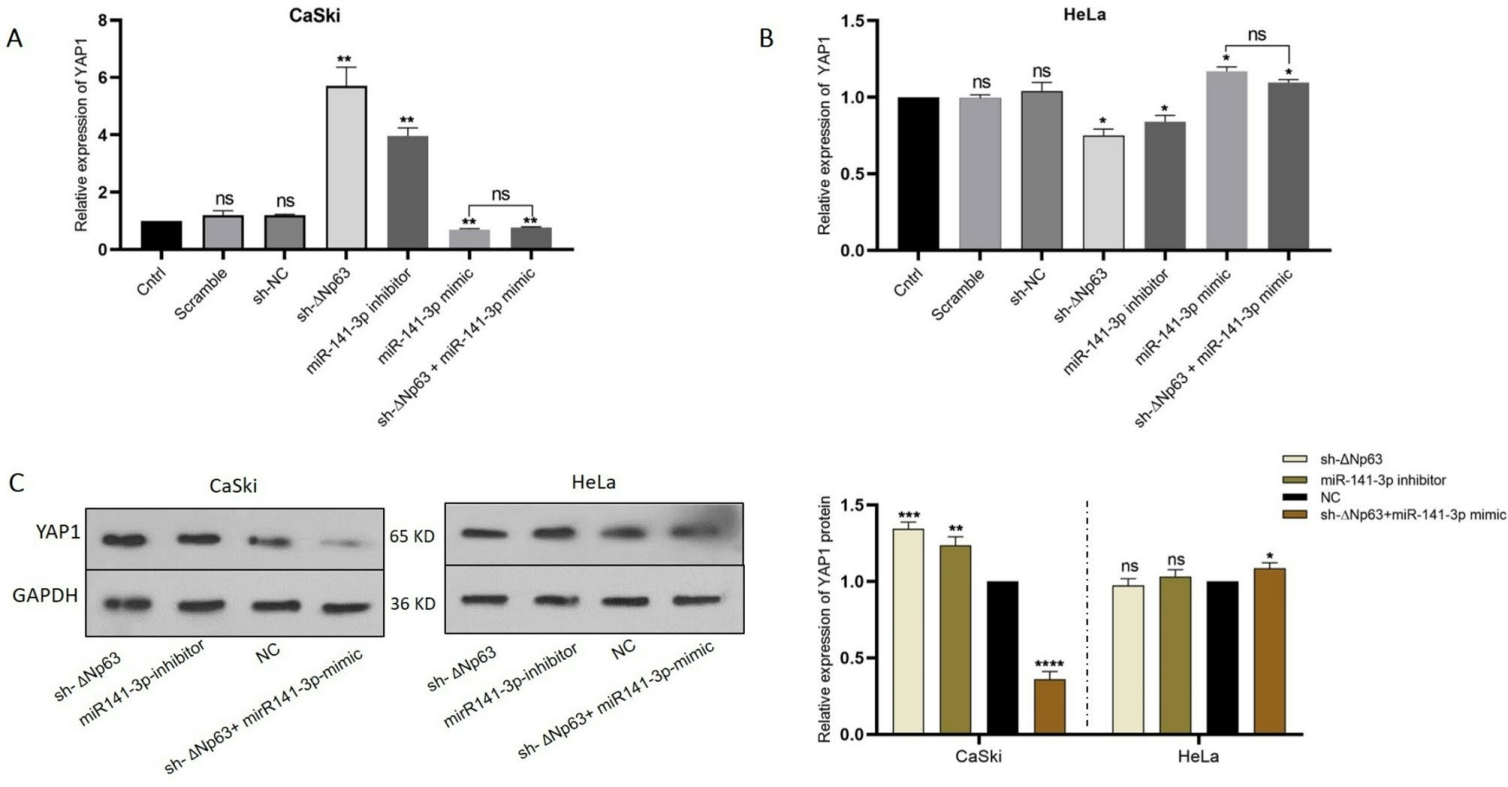
The effect of the ΔNp63-miR-141 axis on YAP1 expression. **A**,** B** YAP1 expression was evaluated by qRT‒PCR after ΔNp63 knockdown, miR-141 down and upregulation, and sh-∆Np63/miR-141-3p mimic co-transfection in CaSki and HeLa cells. Untreated cells (Cntrl), NC-oligos (Scramble), and mock (sh-NC) were used as control groups. Data for transfected groups were compared to those of the untreated control group. **C** Western blot results of YAP1, after transfection with sh-∆Np63, miR-141-3p inhibitor and sh-∆Np63/miR-141-3p mimic. Densitometric analysis of western blot bands was performed using ImageJ software, and the results were normalized to GAPDH. Data are presented as mean ± SD from at least three independent experiments. * *P* < 0. 1, ** *P* < 0.01, *** *P* < 0.001, and **** *P* < 0.0001 indicate a significant difference. Original blots are presented in Supplementary Figs. 1–2.

p63 is known as regulator of a wide variety of microRNAs in different types of cancers^27,33^. An integrative data mining approach identified p63 as a key regulator of microRNAs with different expression profiles in ovarian carcinomas, including miR-141-3p^22^. By using three distinct miRNA target prediction databases (TargetScan, miRmap, and MIRDIP), YAP1 was identified as a possible target of miR-141-3p. To verify the functional relationships among ΔNp63, miR-141-3p, and YAP1, a series of transfection experiments with sh-ΔNp63, the miR-141-3p inhibitors, the mimics, and the sh-ΔNp63/miR-141 mimic were carried out on HeLa and CaSki cells. sh-NC, inh-NC (inhibitor-negative control), and mi-NC (mimic-negative control) were used as negative controls. 48 h and 72 h after transfection, the expression of YAP1, as a downstream target of ΔNp63 and miR-141-3p, was evaluated at the mRNA and protein levels, respectively. First, the expression of YAP1 was significantly increased in CaSki cells after ΔNp63 downregulation (Fig. 5A and C). In contrast, YAP1 was slightly downregulated in HeLa cells in response to ΔNp63 knockdown (Fig. 5B and C). Subsequently, transfection of CaSki cells with inhibitors or mimics led to increased or decreased YAP1 expression, respectively (Fig. 5A and C). In contrast to expectations, transfection of HeLa cells with miR-141-3p inhibitors resulted in a slight increase in YAP1 expression, while transfection with miR-141-3p mimics led to an insignificant decrease in YAP1 expression (Fig. 5B and C). Finally, we aimed to determine whether miR-141-3p acts as a mediator of the effect of ΔNp63 on YAP1. To address this, we performed a rescue assay in the two cell lines using co-transfection with sh-ΔNp63 and miR-141-3p mimic. Modified expression levels of YAP1 due to ΔNp63 knockdown in CaSki and HeLa cells was reversed by miR-141-3p mimic transfection, as depicted in Fig. 5A and B. Data are reported as mean ± SD of three replicates. According to these findings, miR-141-3p functions as an intermediate regulator between ΔNp63 and YAP1. These experimental results were also confirmed by in silico studies, as mentioned previously (see Fig. 1D). Another observation was that while expression of YAP1 in CaSki cells is highly controlled by the ΔNp63-miR-141 axis, YAP1 expression in HeLa cells is not restricted to ΔNp63-miR-14. It is therefore possible that YAP1 is more under the control of other signaling pathways in HeLa cells.The results also showed a positive correlation between p63-miR-141-3p and YAP1 in HeLa cells (*p* < 0.05), whereas a negative correlation was observed in CaSki cells (*p* < 0.01).

### Distinct roles of the ∆Np63-miR-141-3p-YAP1 regulatory axis in the EMT process in different cervical cancer cells

**Fig. 6:**
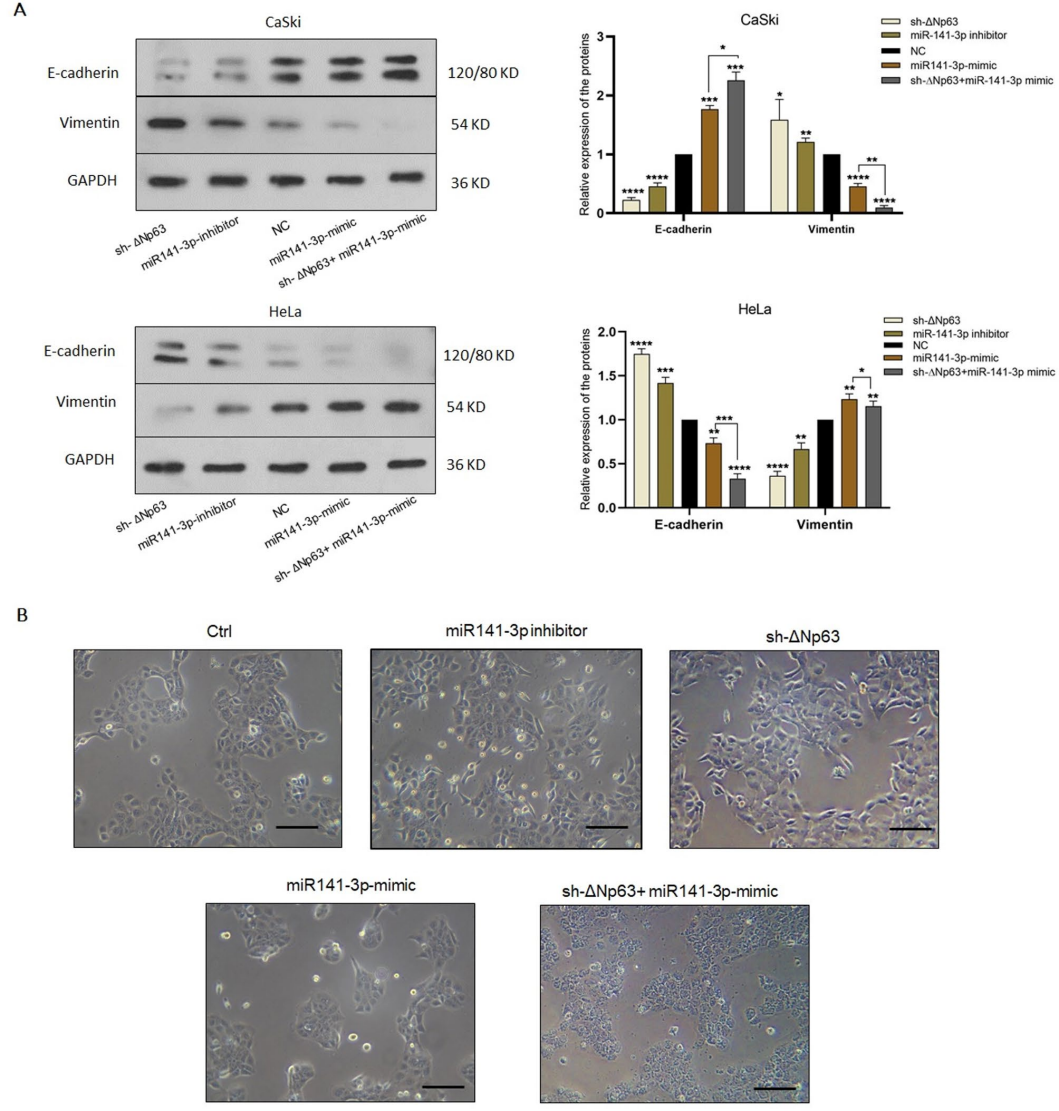
Functional roles of ΔNp63-miR-141-3p axis in the EMT process. **A** Western blot was used to evaluate the expression levels of EMT markers after ΔNp63 silencing and/or miR-141-3p up and downregulation in CaSki and HeLa cells. Densitometric analysis of western blot bands was performed using ImageJ software, and the results were normalized to GAPDH. Data are presented as mean ± SD from at least three independent experiments. **B** The images show untransfected CaSki cells, transfected with sh-ΔNp63, miR-141-3p inhibitor, miR-141-3p mimic and co-transfected with sh-ΔNp63/ miR-141mimic. Compared to other groups, CaSki cells transfected with sh-ΔNp63 and miR-141-3p inhibitor, exhibited a spindle-like morphology and less cell-cell junctions. Magnification ×10, scale bar 200 μm. Original blots are presented in Supplementary Figs. 3–8.

To investigate the influence of this regulatory axis on the progression of EMT, we analysed the epithelial E-cadherin and mesenchymal Vimentin markers by Western blotting. Figure 6A shows that CaSki cells transfected by sh-∆Np63- and miR-141-3p inhibitor had less E-cadherin and more Vimentin compared to control groups. As expected, transfection of the miR-141-3p mimic- and sh-∆Np63/miR-141-3p mimic reversed the expression patterns of E-cadherin and Vimentin (Fig. 6A). These results support the findings of the migration and invasion experiments in CaSki cells. Consistent with the reduced E-cadherin expression, morphological changes in CaSki cells 3 days after sh-∆Np63 transfection revealed an elongated spindle shape and weakened cell-cell adhesion (Fig. 6B). Transfected cells with the sh-∆Np63/miR-141-3p mimic lost their spindle-like morphology (Fig. 6B). In the more aggressive HeLa cells, E-cadherin and Vimentin markers were differentially expressed compared to CaSki cells. Downregulation of ∆Np63 and miR-141-3p, which are positive regulators of YAP1 in HeLa cells, led to an increase in the level of E-cadherin and a decrease in the level of Vimentin. As expected, the results of the miR-141-3p mimic and sh-∆Np63/1miR-141-3p mimic co-transfections reversed the results (Fig. 6A). From morphological perspective, CaSki cells exhibited significant morphological changes after transfections compared to control gruops (Fig. 6B), whereas no significant morphological changes were observed in HeLa cells after transfection, perhaps because these cells are more aggressive and have a naturally spindle shape.

## Discussion

The distinct roles of p63 isoforms in cancer progression have been reported by independent studies. ΔNp63 acts as a potent oncogene in various SCCs, and its overexpression promotes cancer progression by enhancing cell proliferation and inhibiting apoptosis^34–37^. Regarding the role of ΔNp63 in cancer progression, contradictory results have been published until now. Depending on the cancer type, loss of ΔNp63 expression may lead to decreased cell proliferation by inhibiting cell cycle progression, cellular senescence, or apoptosis, as observed in keratinocytes and SCCs^38–40^. Other groups reported that high expression of ∆Np63 prevents migration, invasion, and EMT in different cancers, such as urothelial neoplasms and bladder, prostate, and squamous carcinomas^17,27,33,41^. Thus, ΔNp63 might play a central role in triggering tumorigenesis by enhancing proliferation or preventing apoptosis, while its suppression is required for EMT progression and metastasis^42^. ΔNp63 has also been identified as a key regulator of microRNAs by directly regulating their expression and influencing diverse cellular processes, including the EMT. For example, ΔNp63 promotes the expression of EMT-regulating microRNAs such as miR-205 and miR-200 family members by directly binding to their promoters^22,27,33^. Our findings introduce ΔNp63 as a positive regulator of miR-141-3p, a member of the miR-200 family, in both CaSki and HeLa cells. These observations confirm the conclusion of Knouf et al. on p63 and miR-200 family in ovarian cancer^22^. Loss of miR-141 is associated with poor prognosis in prostate and hepatocellular carcinomas via the modulation of EMT and angiogenesis regulatory cascades^30,43^. Instead, pro-metastatic roles of miR-141 have also been reported in endometrial and nasopharyngeal cancers^23,44^. Independent studies have shown that miR-141-3p acts as an intermediate regulator of the EMT progression in several cancers. For example, two non-coding RNAs, SNHG15 and circATRNL1, have been shown to promote EMT through sponging miR-141-3p^32,45^. We showed that the ΔNp63-miR-141-3p axis has pro- and anti-metastatic functions in ADC HeLa and SCC CaSki cells, respectively. Rescue assays revealed that sh-ΔNp63/miR-141-3p mimic co-transfection could rescue cells from the effect of ΔNp63 knockdown on the regulation of migration, invasion, and EMT of both cervical cancer cell lines.

As evidenced by the in silico network, the activation of p63 serves as a catalyst for the transcription of the microRNA family miR-200 (Fig. 1D). Additionally, p63 can influence the EMT by specifically targeting YAP1 and ZEB1 through direct targeting of miR-141-3p. In silico analysis also revealed a negative correlation between p63 and YAP1 expression levels in ADC and SCC tumor samples (Fig. 1A and B). Different experiments have shown that in comparison to SCC, ADC is more aggressive and has a greater metastasis rate and poorer prognosis^3,46–50^. In ADC HeLa cells, the expression of YAP1, a key promoter of EMT, was dramatically greater than that in CaSki cells, as confirmed by qRT‒PCR. In HeLa cells, a slight decrease in the level of YAP1 expression in response to ∆Np63 and miR-141-3p inhibition demonstrated that YAP1 is not fully under the control of the ΔNp63-miR-141-3p regulatory axis. In contrast, in CaSki cells (SCC cells with high expression levels of ΔNp63 and low expression of YAP1), downregulation of the ΔNp63-miR-141-3p axis resulted in YAP1 upregulation and enhanced the migration and invasion capacities of these cells. According to in silico findings, the expression level of YAP1 in SCC samples is slightly lower than that in ADC samples. This observation is supported by previous studies indicating the crucial role of YAP1 in promoting EMT, a vital step in the progression and metastasis of tumors^7,8^. By regulating the expression of key factors involved in EMT, YAP1 facilitates the transformation of epithelial cells to a mesenchymal phenotype, enabling them to escape their normal tissue environment and acquire the ability to invade and metastasize^51–53^. Consequently, the high expression of YAP1 in ADC, along with the concurrent decrease in the expression of p63, substantiates its significant invasive properties. Notably, miR-141-3p can target both ZEB1 and YAP1. Furthermore, the coactivation of ZEB1 and YAP1 has been demonstrated to promote plasticity in EMT and metastasis in pancreatic cancer^52^. This conclusion can be drawn based on the established network, indicating that miR-141 can influence EMT by targeting ZEB1 and YAP1. Taken together, these results indicate that YAP1 acts as a key oncogene in promoting tumorigenic properties in both of studied cervical cancer cell lines, whereas the ∆Np63–miR-141-3p regulatory axis exhibits distinct, context-dependent roles in different cell lines.

Although we assessed YAP1 expression levels in our experimental conditions, it is important to emphasize that YAP1 activity is not solely determined by its expression. YAP1 is regulated post-translationally through phosphorylation, particularly at Ser127^54,55^, which promotes its cytoplasmic retention and inactivation. In contrast, dephosphorylated YAP1 translocates to the nucleus, where it functions as a transcriptional co-activator of target genes involved in proliferation and survival. Therefore, YAP1’s functional status depends not only on its abundance but also on its phosphorylation state and subcellular localization. These factors should be taken into account when interpreting the biological significance of YAP1 in our model.

The increased migration and invasion abilities and decreased proliferation rate of CaSki cells in response to ∆Np63 downregulation were also interesting results of this study, which implies that in some epithelial cancer types, decreased proliferative ability is necessary for triggering migration, invasion, and EMT, as reported previously^42^. Thus, we hypothesized that the ∆Np63-miR-141-3p-YAP1 regulatory axis may play a dual role in EMT. Notably, the specific roles of ∆Np63 and miR-141-3p in migration, invasion, and EMT can vary depending on the cellular context, tissue type, and presence of other molecular players.

**Fig. 7:**
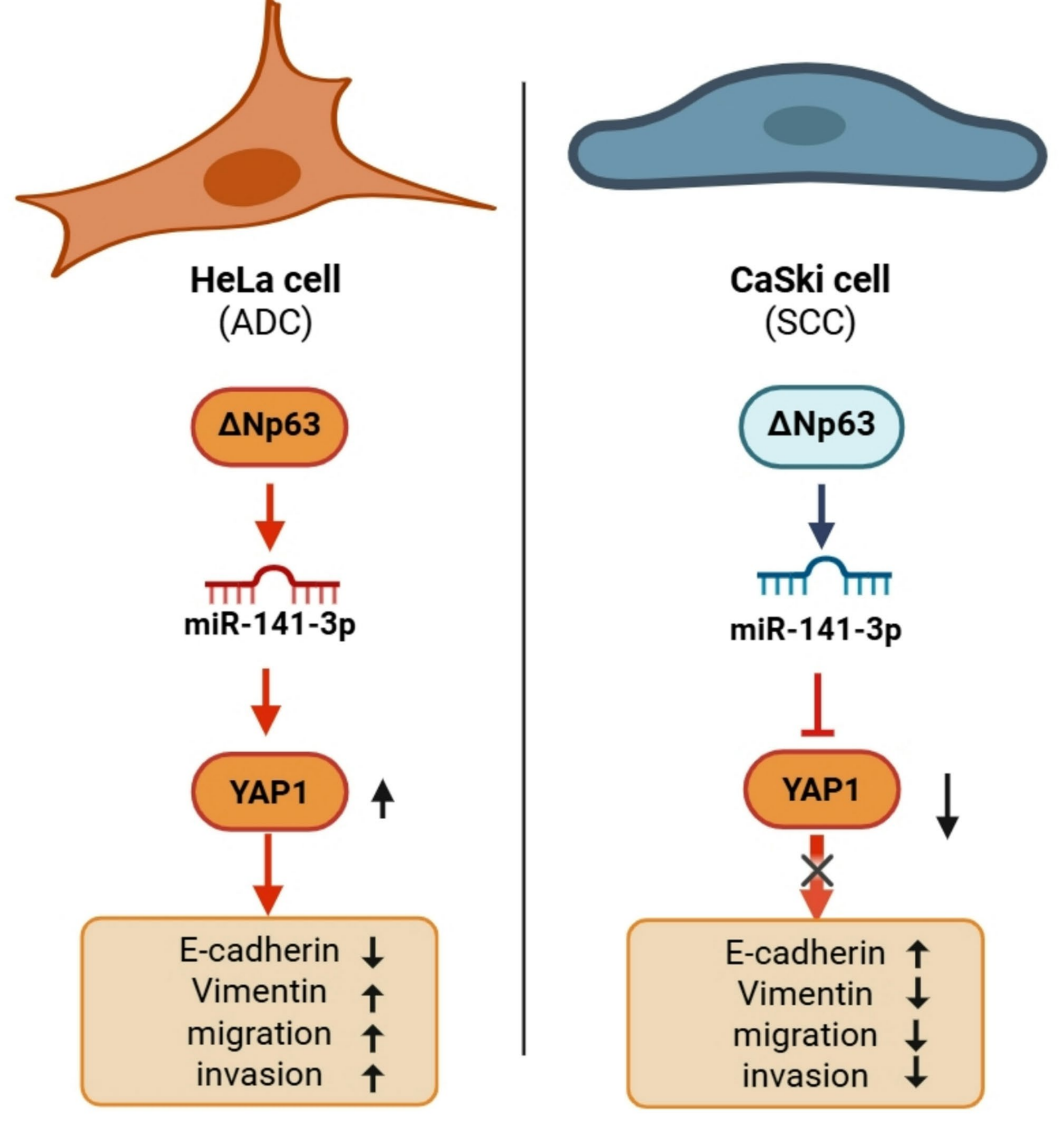
Schematic illustration of the ΔNp63–miR-141-3p–YAP1 axis in HeLa (ADC) and CaSki (SCC) cervical cancer cell lines. This regulatory axis promotes EMT, migration, and invasion in HeLa cells but suppresses these processes in CaSki cells through differential YAP1 modulation.

Overall, these findings suggested a potential regulatory axis in which p63, an upstream transcription factor, could influence the expression of miR-141-3p, and in turn, miR-141-3p could modulate the expression of YAP1. Our results demonstrated that in HeLa and CaSki cells, YAP-1 has diverse functional interactions with p63, and miR-141-3p could act as a fine-tuning partner in the control of YAP-1 expression in this regulatory cascade (see Fig. 7).

## Conclusion

This study investigates the interplay between ΔNp63, miR-141-3p, and YAP1 in the progression of cervical cancer. The findings reveal a complex and cell line-dependent regulatory axis involving ΔNp63, miR-141-3p, and YAP1, which significantly influences epithelial–mesenchymal transition (EMT) and metastatic potential. Our results underscore the pivotal role of this axis in modulating cervical cancer progression; however, its function appears to be highly dependent on the specific molecular and phenotypic characteristics of each cancer subtype. Further research is warranted to elucidate the precise molecular mechanisms governing this regulatory network and to assess its viability as a therapeutic target in cervical cancer.

## Electronic supplementary material

Below is the link to the electronic supplementary material.

Supplementary Material 1

## Data Availability

All data can be available upon request from Majid Sadeghizadeh, Tarbiat Modares University, sadeghma@modares.ac.ir.
