# Supplementary material for: Dual functions of the ΔNp63-miR-141-3p-YAP1 regulatory axis in cervical cancer progression are dependent on histological subtype
Source: Sci Rep. 2025 Jul 2;15:23155. doi: 10.1038/s41598-025-07237-6 (PMC12222551; doi:10.1038/s41598-025-07237-6)
Supplement: Supplementary file 1 — Supplementary Material 1 [file 41598_2025_7237_MOESM1_ESM.pdf]

Dual functions of the ΔNp63-miR-141-3p-YAP1 regulatory axis in cervical cancer progression are dependent on histological subtype

Somayeh Panahi-Moghadam[1], Majid Sadeghizadeh[1*], Shirin Farivar[2*], Faezeh Vakhshiteh[3]

*** Correspondences:**

Majid Sadeghizadeh, Department of Molecular Genetics, Faculty of Biological Sciences, Tarbiat Modares University, Tehran, Iran Tel: (+9821)82884409, Fax: (+9821)82884717

Email: sadeghma@modares.ac.ir

Shirin Farivar, Department of Cell and Molecular Biology, Faculty of Life Sciences and Biotechnology, Shahid Beheshti University, Tehran, Iran Tel: (+9821)29905932

Email: s_farivar@sbu.ac.ir

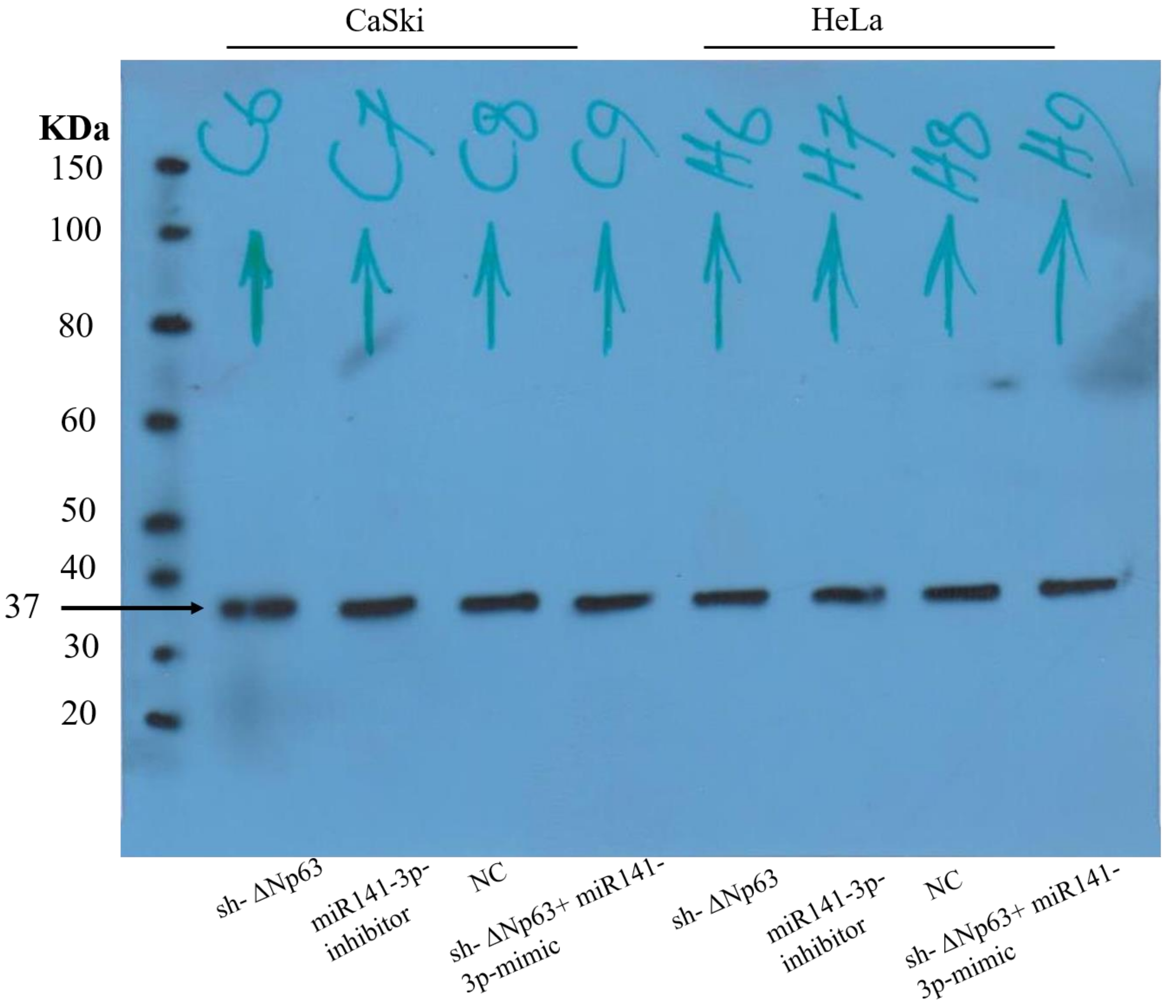

Fig 1. Western blotting of the **GAPDH**. Lane1: protein molecular weight marker, Lane2-5 CaSki cells; Lane 2: ΔNp63 knockdown, Lane3: miR-141-3p knockdown, Lane4: Negative control, Lane5: One-step co-transfection (ΔNp63 knockdown and miR-141-3p overexpression); Lane 6-10 HeLa cells; Lane 2: ΔNp63 knockdown, Lane3: miR-141 knockdown, Lane4: Negative control, Lane5: One-step co-transfection (ΔNp63 knockdown and miR-141-3p overexpression).

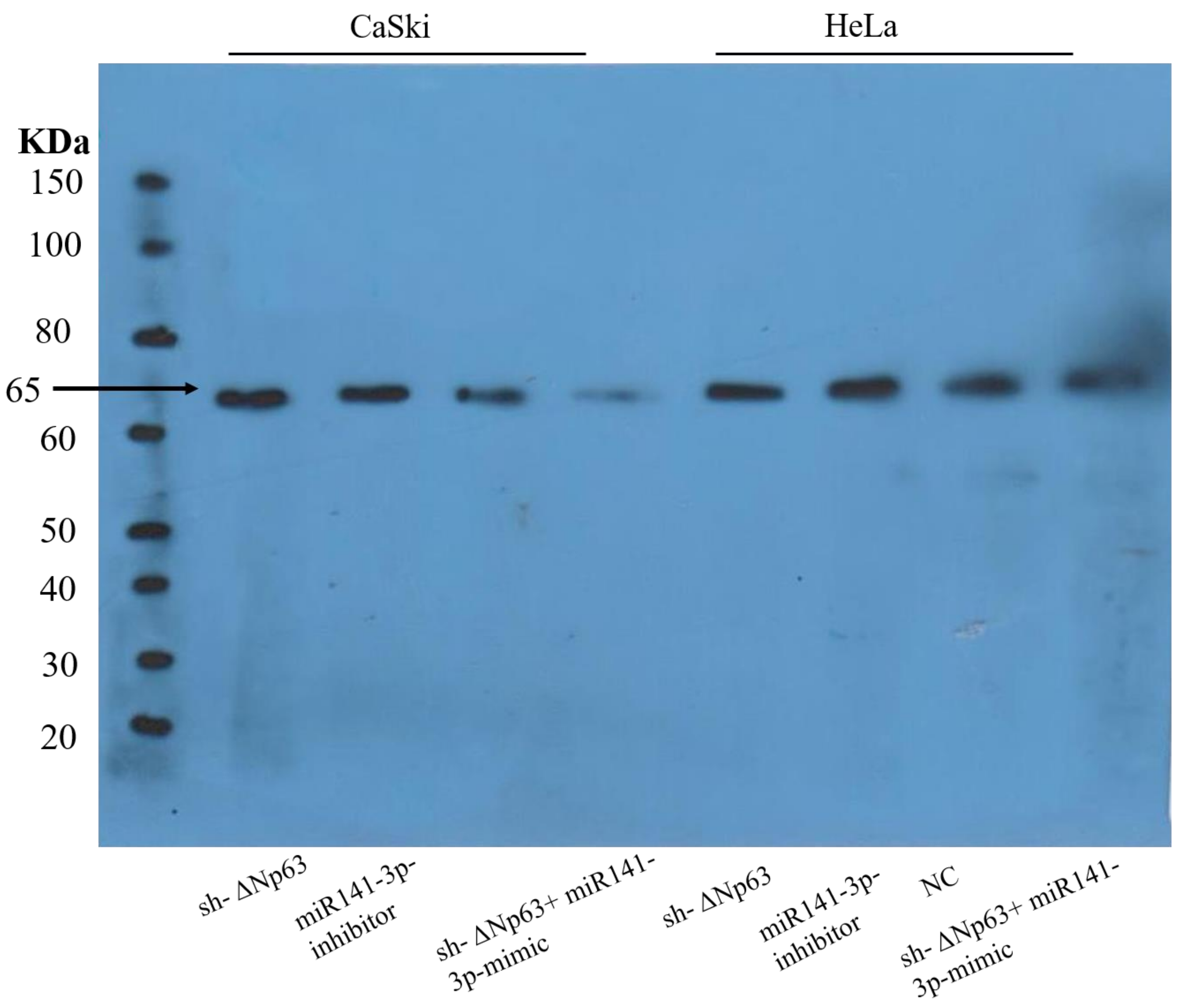

Fig 2. Western blotting of the **YAP1**. Lane1: protein molecular weight marker, Lane2-5 CaSki cells; Lane 2: ΔNp63 knockdown, Lane3: miR-141-3p knockdown, Lane4: Negative control, Lane5: One-step co-transfection (ΔNp63 knockdown and miR-141-3p overexpression); Lane 6-10 HeLa cells; Lane 2: ΔNp63 knockdown, Lane3: miR-141 knockdown, Lane4: Negative control, Lane5: One-step co-transfection (ΔNp63 knockdown and miR-141-3p overexpression).

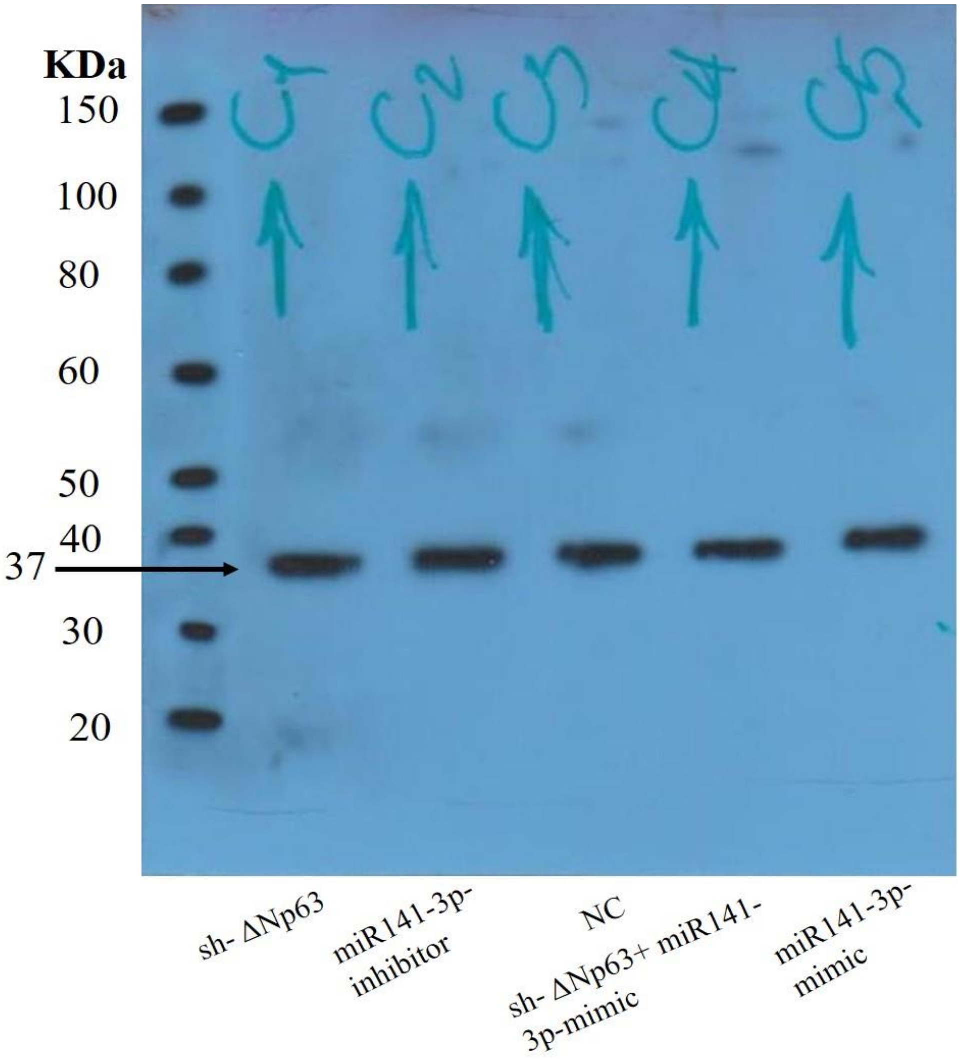

Fig 3. Western blotting of the **GAPDH**. Lane1: protein molecular weight marker, Lane2-6 CaSki cells; Lane 2: ΔNp63 knockdown, Lane3: miR-141-3p knockdown, Lane4: Negative control, Lane5: One-step co-transfection (ΔNp63 knockdown and miR-141-3p overexpression); Lane 6: miR-141-3p overexpression

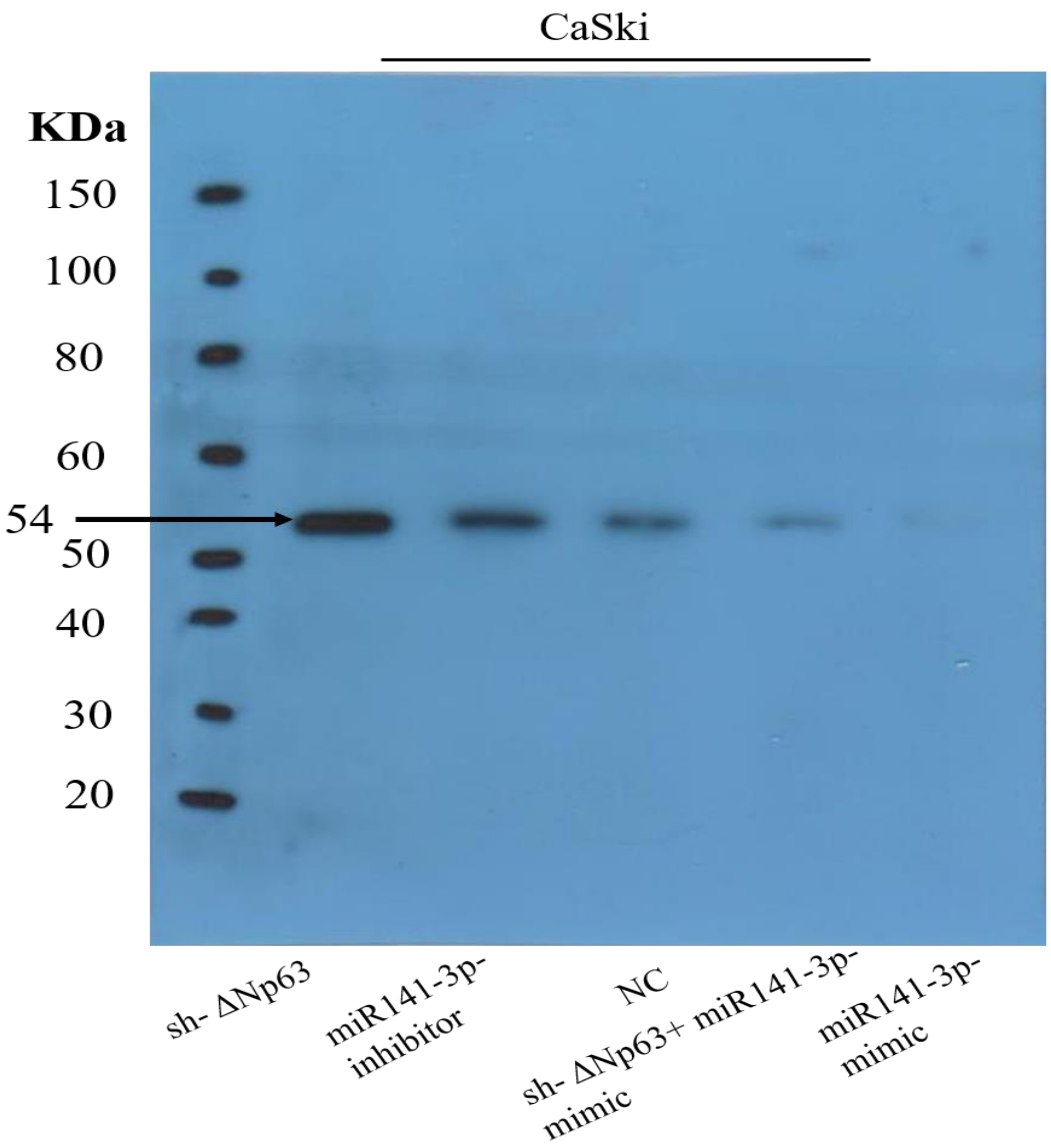

Fig 4. Western blotting of the **Vimentin**. Lane1: protein molecular weight marker, Lane2-6 CaSki cells; Lane 2: ΔNp63 knockdown, Lane3: miR-141-3p knockdown, Lane4: Negative control, Lane5: One-step co-transfection (ΔNp63 knockdown and miR-141-3p overexpression); Lane 6: miR-141-3p overexpression.

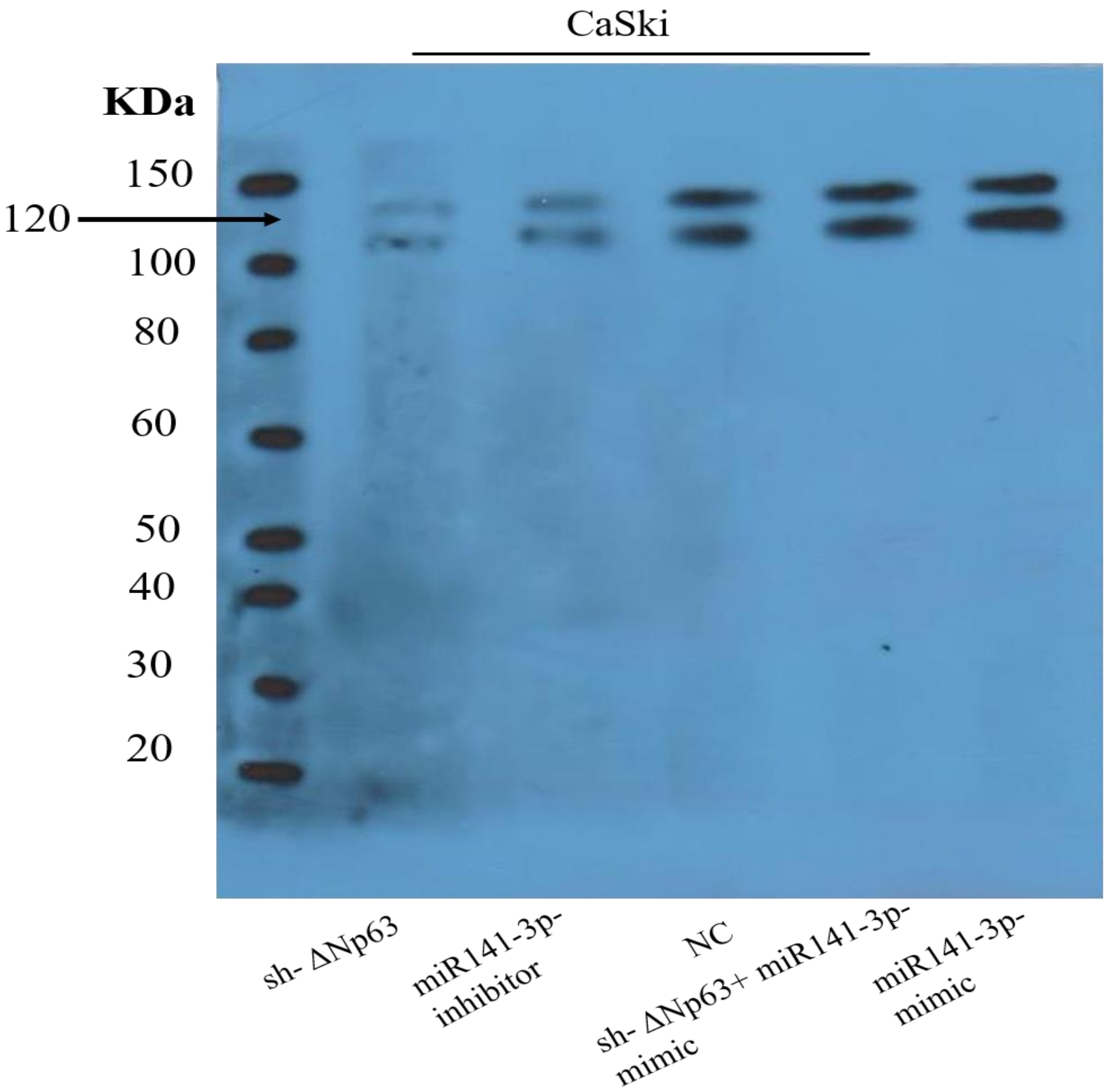

Fig 5. Western blotting of the **E-Cadherin**. Lane1: protein molecular weight marker, Lane2-6 CaSki cells; Lane 2: ΔNp63 knockdown, Lane3: miR-141-3p knockdown, Lane4: Negative control, Lane5: One-step co-transfection (ΔNp63 knockdown and miR-141-3p overexpression); Lane 6: miR-141-3p overexpression.

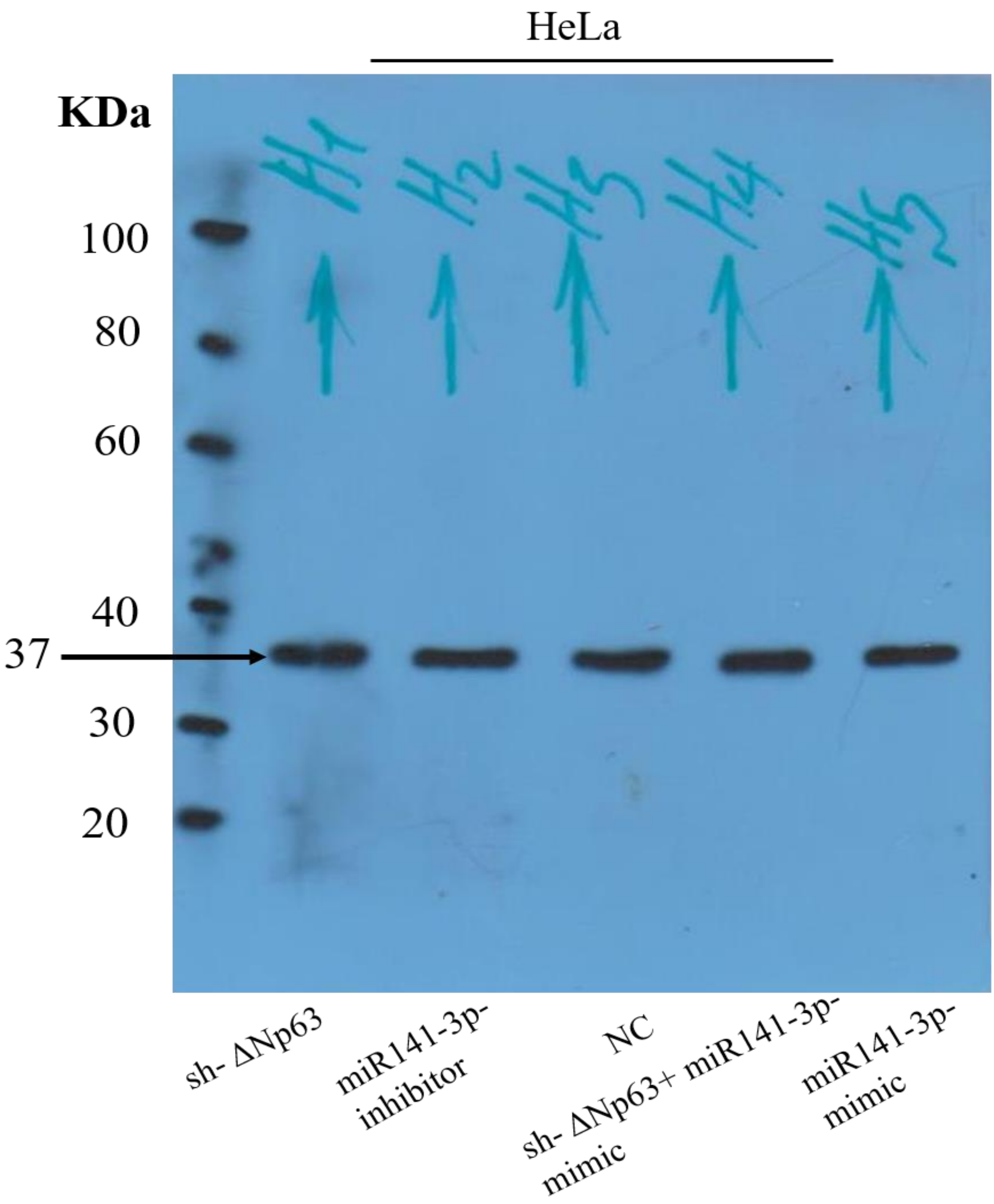

Fig 6. Western blotting of the **GAPDH**. Lane1: protein molecular weight marker, Lane2-6 HeLa cells; Lane 2: ΔNp63 knockdown, Lane3: miR-141-3p knockdown, Lane4: Negative control, Lane5: One-step co-transfection (ΔNp63 knockdown and miR-141-3p overexpression); Lane 6: miR-141-3p overexpression.

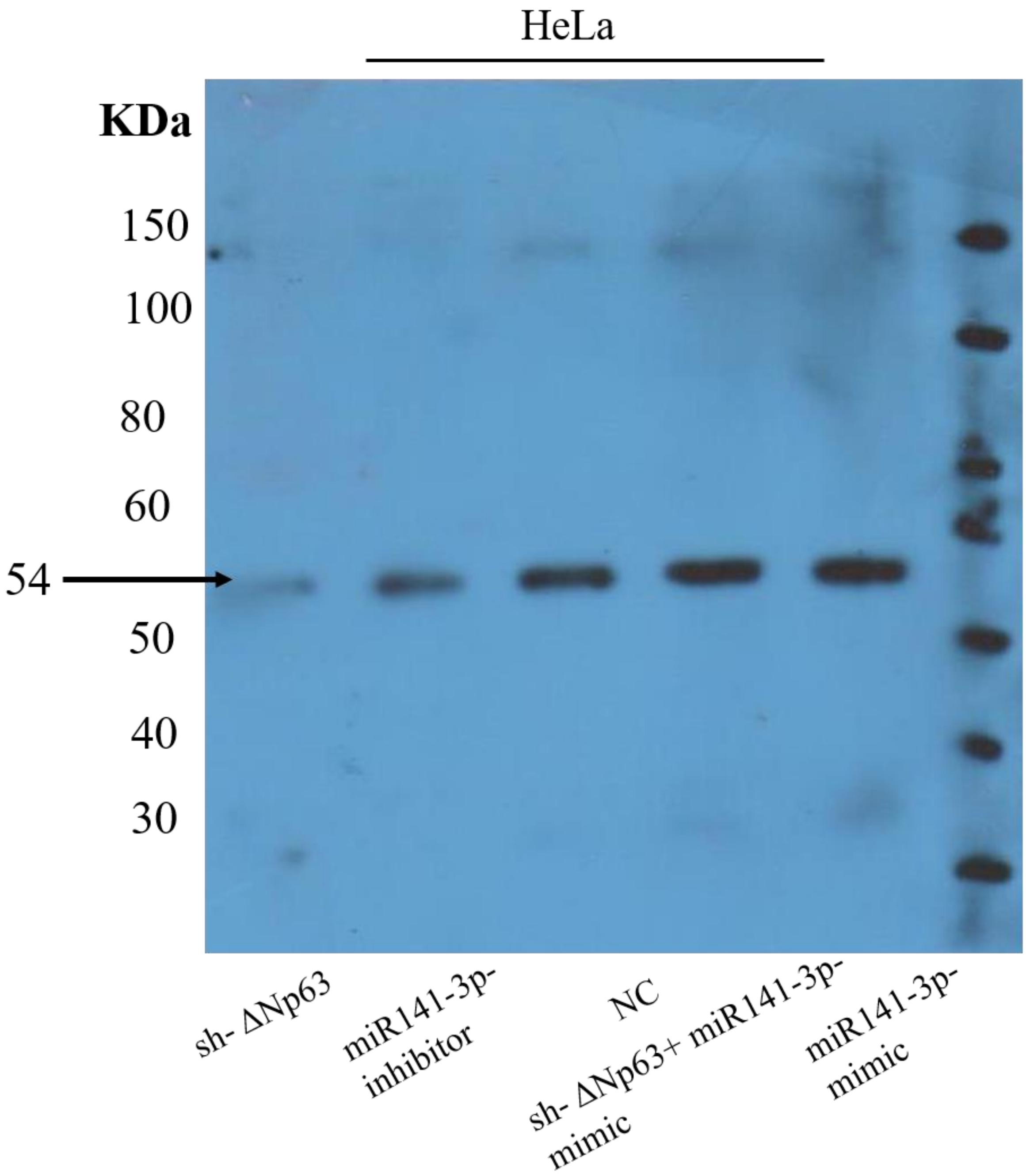

Fig 7. Western blotting of the **Vimentin**. Lane1: protein molecular weight marker, Lane2-6 HeLa cells; Lane 2: ΔNp63 knockdown, Lane3: miR-141-3p knockdown, Lane4: Negative control, Lane5: One-step co-transfection (ΔNp63 knockdown and miR-141-3p overexpression); Lane 6: miR-141-3p overexpression.

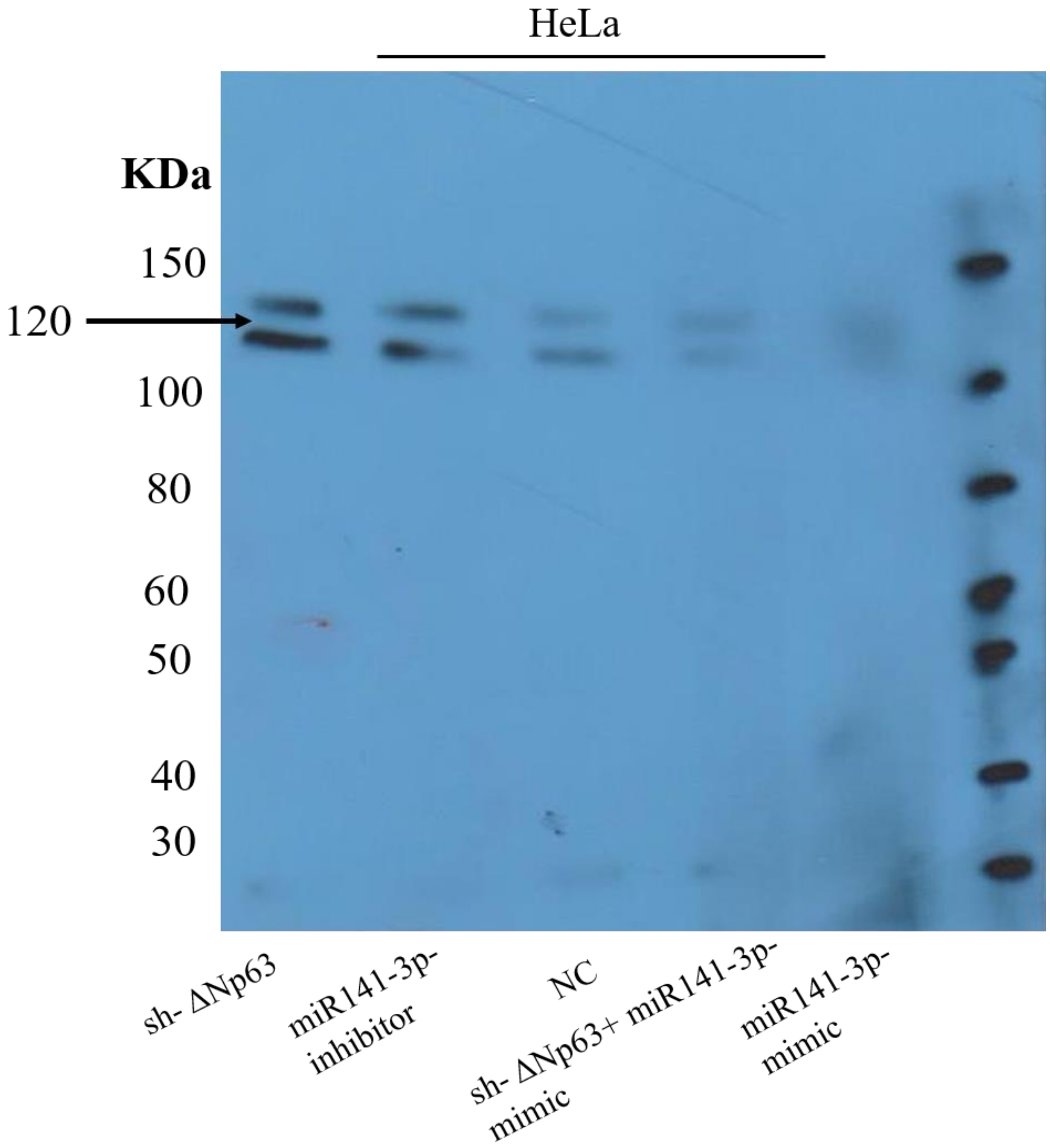

Fig 8. Western blotting of the **E-Cadherin**. Lane1: protein molecular weight marker, Lane2-6 HeLa cells; Lane 2: ΔNp63 knockdown, Lane3: miR-141-3p knockdown, Lane4: Negative control, Lane5: One-step co-transfection (ΔNp63 knockdown and miR-141-3p overexpression); Lane 6: miR-141-3p overexpression.
